# The non-receptor tyrosine phosphatase type 14 blocks caveolin-1-enhanced cancer cell metastasis

**DOI:** 10.1038/s41388-020-1242-3

**Published:** 2020-03-09

**Authors:** Natalia I. Díaz-Valdivia, Jorge Díaz, Pamela Contreras, América Campos, Victoria Rojas-Celis, Renato A. Burgos-Ravanal, Lorena Lobos-González, Vicente A. Torres, Viviana I. Perez, Balz Frei, Lisette Leyton, Andrew F. G. Quest

**Affiliations:** 1 Cellular Communication Laboratory, Center for studies on Exercise, Metabolism and Cancer (CEMC), Advanced Center for Chronic Diseases (ACCDiS), Faculty of Medicine, Universidad de Chile, Santiago, Chile; 2 Institute for Research in Dental Science, Faculty of Dentistry, Universidad de Chile, Santiago, Chile; 3 Fundación Ciencia & Vida, Santiago, Chile; 4 Department of Biochemistry and Biophysics, Linus Pauling Institute, Oregon State University, Corvallis, OR USA

**Keywords:** Metastasis, Mechanisms of disease, Phosphorylation

## Abstract

Caveolin-1 (CAV1) enhanced migration, invasion, and metastasis of cancer cells is inhibited by co-expression of the glycoprotein E-cadherin. Although the two proteins form a multiprotein complex that includes β-catenin, it remained unclear how this would contribute to blocking the metastasis promoting function of CAV1. Here, we characterized by mass spectrometry the protein composition of CAV1 immunoprecipitates from B16F10 murine melanoma cells expressing or not E-cadherin. The novel protein tyrosine phosphatase PTPN14 was identified by mass spectrometry analysis exclusively in co-immunoprecipitates of CAV1 with E-cadherin. Interestingly, PTPN14 is implicated in controlling metastasis, but only few known PTPN14 substrates exist. We corroborated by western blotting experiments that PTPN14 and CAV1 co-inmunoprecipitated in the presence of E-cadherin in B16F10 melanoma and other cancer cells. Moreover, the CAV1(Y14F) mutant protein was shown to co-immunoprecipitate with PTPN14 even in the absence of E-cadherin, and overexpression of PTPN14 reduced CAV1 phosphorylation on tyrosine-14, as well as suppressed CAV1-enhanced cell migration, invasion and Rac-1 activation in B16F10, metastatic colon [HT29(US)] and breast cancer (MDA-MB-231) cell lines. Finally, PTPN14 overexpression in B16F10 cells reduced the ability of CAV1 to induce metastasis in vivo. In summary, we identify here CAV1 as a novel substrate for PTPN14 and show that overexpression of this phosphatase suffices to reduce CAV1-induced metastasis.

## Introduction

Caveolin-1 (CAV1) is an integral membrane protein that plays a dual role in tumor progression. On the one hand, CAV1 protein and mRNA levels are decreased in transformed fibroblasts, in breast, and colon cancer cell lines [1, 2], and re-expression of CAV1 in colon cancer cells reduces subcutaneous tumor formation in immunosuppressed nude mice [3]. On the other hand, CAV1 acts as a promoter of metastasis in later stages of cancer, where enhanced expression of CAV1 favors the malignant phenotype and correlates with an increase in cell migration, metastasis [4–8], and multidrug resistance [9]. The two first mentioned roles of CAV1 are affected by the presence of the glycoprotein E-cadherin, which enhances CAV1 function as a tumor suppressor and blocks the ability of CAV1 to promote metastasis in vivo. The tumor suppressor function of CAV1 in the presence of E-cadherin is associated with the formation of a multiprotein complex that recruits β-catenin to the plasma membrane [10], thereby decreasing β-catenin/Tcf-Lef dependent expression of the protein survivin and cyclooxygenase-2, which promote cell survival [11, 12]. However, the precise mechanism by which E-cadherin suppresses the ability of CAV1 to promote metastasis is unknown.

The ability of CAV1 to promote migration of metastatic cells is associated with increased activation of the small GTPases Rab-5 and Rac-1 [13]. For instance, in the metastatic breast cancer cell line MDA-MB-231, CAV1-dependent activation of Rac-1 is observed in spreading assays [4]. Moreover, CAV1-mediated activation of Rab-5 in metastatic cells is required to increase cell migration in a Rac-1-dependent manner [13, 14].

CAV1 is subjected to post-translational modifications relevant to protein function, such as phosphorylation on tyrosine-14 (pY14) by the non-receptor protein tyrosine kinases Src, Fyn, and Abl. This may occur in response to a large variety of stimuli, such as insulin, ultraviolet radiation, hydrogen peroxide, hyperosmolarity, and hemodynamic shear stress [15–23]. Upon Y14 phosphorylation, CAV1 binds to and inactivates the C-terminal Src kinase (Csk), thereby increasing Src activity [24], which is required for CAV1 to enhance cell migration [4, 25–28]. In metastatic breast cancer and melanoma cells, CAV1 phosphorylation on Y14 increases cell migration by promoting focal adhesion turnover, polarization, persistency, speed, and directionality of migration. All these functions of CAV1 are blocked either pharmacologically using Src family kinase inhibitors or by introducing a non-phosphorylatable CAV1(Y14F) mutation, which also completely blocks the metastasis promoting role of CAV1 [4, 7].

The relationship between CAV1 and phosphatases has not been extensively studied, although possible candidates that dephosphorylate CAV1 pY14 have been described [29]. In none of these cases, however, has a connection to the control of CAV1-enhanced migration, invasion, and metastasis been established. For example, hydrogen peroxide-induced oxidative stress promotes the phosphorylation of the tyrosine phosphatase SHP-2 in astrocytes, where it forms a multiprotein complex with the CAV1 wild type protein, but not with a non-phosphorylatable CAV1(Y14A) mutant. The overexpression of the tyrosine phosphatase 1B (PTP1B) decreases CAV1 pY14 levels in COS-7 cells, while the inhibition of PTP1B induces CAV1 Y14 phosphorylation in vitro and in situ [30].

On the other hand, the non-receptor tyrosine phosphatase type 14 (PTPN14), also known as Pez, PTP36, or PTPD2, regulates cell–cell and cell–matrix adhesion, cell migration, and growth [31]. In colorectal cancer cells, PTPN14, dephosphorylates 130Cas pY28, a site phosphorylated by Src, to decrease migration, colony formation, and anchorage-independent growth [31]. Moreover, the ability of PTPN14 to suppress breast cancer cell metastasis in experimental animal models was linked to a reduction in protein trafficking through the secretory pathway. In this context, RIN (Ras and Rab interactor 1) and PKCδ (Protein kinase Cδ) were identified as PTPN14 binding partners and targets [32]. Thus, while PTPN14 has previously been linked to the inhibition of metastasis, a role for CAV1 in this context has not been reported.

Here, we identified the mechanism by which E-cadherin suppresses CAV1-enhanced metastasis. We show that tyrosine phosphorylation of Y14-CAV1 is reduced in E-cadherin expressing cells. Moreover, we identify PTPN14 as a phosphatase that is selectively detected in CAV1 immunoprecipitates from cells expressing E-cadherin and presence of PTPN14 in CAV1/E-cadherin complexes is associated with reduced phosphorylation of Y14-CAV1. Finally, we show that PTPN14 overexpression blocks CAV1-enhanced migration, invasion, and metastasis of cancer cells due to its catalytic activity.

## Results

### E-cadherin expression decreases CAV1 phosphorylation on tyrosine-14 in metastatic cancer cell lines

**Fig. 1:**
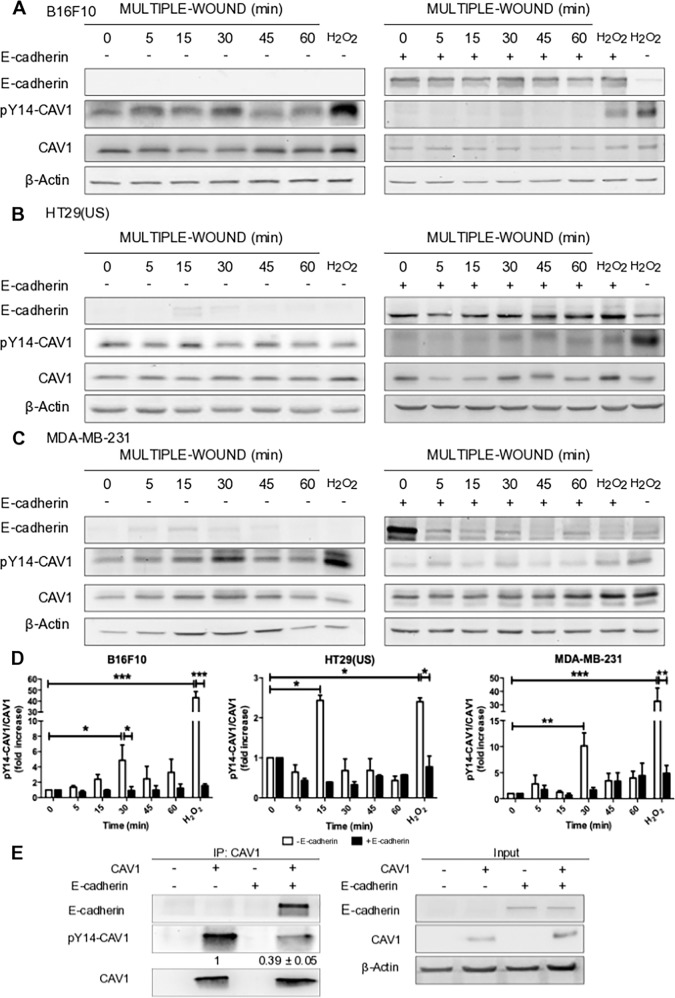
E-cadherin expression decreases caveolin-1 phosphorylation on tyrosine 14 in metastatic cancer cell lines. **a** B16F10(CAV1), B16F10(CAV1-Ecadherin), **b** HT29(US)(CAV1), HT29(US)(CAV1-Ecadherin), or **c** MDA-MB-231(shC), MDA-MB-231(shC-Ecadherin) cells (6 × 10^5^) were seeded in 6 cm dishes and grown to confluency. Then multiple wounds covering at least 50% of the total monolayer surface were introduced with a steel comb. Subsequently, cell monolayers were washed with PBS and either serum free medium (time 0) or medium containing 3% serum was added to stimulate migration for different times (5, 15, 30, 45, and 60 min). Cells incubated with 5 mM H_2_O_2_ for 20 min were included in each set of experiments as a positive control for CAV1 phosphorylation. Proteins (50 µg) were then separated by SDS-PAGE, transferred to nitrocellulose and the presence of E-cadherin, Caveolin-1 phosphorylated on Tyr-14 (pY14-CAV1), caveolin-1 (CAV1), and β-actin were detected using specific antibodies. Blots representative of results obtained in three independent experiments are shown. **d** Results obtained by western blotting were quantified by scanning densitometry. The graphs show the means from 3 independent experiments (mean ± SEM) of the ratio pY14-CAV1/CAV1 normalized to the control condition (time 0). The white bars represent results from cells that do not express E-cadherin while the black bars are from E-cadherin expressing cells. Statistically significant differences are indicated ***p < 0.001, **p < 0.01, *p < 0.05. **e** CAV1 was immunoprecipitated from protein extracts containing 2 mg of total protein obtained from B16F10(Mock), B16F10(CAV1), B16F10(Mock-Ecadherin) or B16F10(CAV1-Ecadherin) cells using a polyclonal antibody against CAV1 (2.5 μg/condition). Immunoprecipitated CAV1, Y14 phosphorylation of CAV1, and co-immunoprecipitated E-cadherin were detected by immunoblotting. An immunoblot with the total protein input in each experiment is included in the panel to the right. Western blots representative of results obtained in 3 independent experiments are shown. Numerical values shown beneath lanes in the left panel are means of values from 3 independent experiments (mean ± SEM) of the ratio pY14-CAV1/CAV1 for CAV1 phosphorylation in the presence of E-cadherin normalized to the control condition without E-cadherin.

CAV1 phosphorylation on Y14 is essential to promote migration/invasion and metastatic cells have elevated levels of CAV1 pY14 in comparison to non-metastatic cancer cells [7, 33]. Alternatively, the expression of E-cadherin blocks CAV1-enhanced metastasis in vivo [5]. These observations led us to ask whether the expression of E-cadherin modulated Y14-CAV1 phosphorylation. To test this possibility, migration was induced in confluent cell monolayers by introducing multiple wounds and CAV1 pY14 was evaluated in mouse melanoma B16F10 and human colon adenocarcinoma HT29(US). These cell lines were stably transfected with the plasmid pLacIOP-CAV1 that permits IPTG-inducible expression of CAV1. For the human breast cancer cells MDA-MB-231, which express CAV1 endogenously, levels of this protein were reduced using specific short hairpin constructs. All three metastatic cell lines express very low levels of E-cadherin (Supplementary Fig. 1a). We observed by Western blotting an increase in CAV1 pY14, 30 min after multiple wounding of B16F10 (Fig. 1a, left panel), after 15 min for HT29(US) cells (Fig. 1b, left panel), and after 30 min for MDA-MB-231 cell monolayers (Fig. 1c, left panel). The three cell lines were then transfected with the plasmid pBATEM2 to augment the expression of E-cadherin as previously described [5]. The presence of E-cadherin reduced phosphorylation of CAV1 on Y14 following monolayer wounding (Fig. 1a–c, right panels). Therefore, E-cadherin expression in the three metastatic cell lines analyzed here lead to a significant decrease in CAV1 pY14 following induction of migration (Fig. 1d).

We have previously observed that CAV1 and E-cadherin co-immunoprecipitate and colocalize in cells [5, 10]. However, whether E-cadherin presence in E-cadherin/CAV1 complexes might reduce Y14 phosphorylation levels was not known. Thus, we immunoprecipitated CAV1 in E-cadherin expressing B16F10 cells [B16F10(CAV1-E-cadherin)] and confirmed that E-cadherin co-immunoprecipitated with CAV1. We also observed that CAV1 pY14 levels were reduced by 60% in immunoprecipitates from cells that express E-cadherin (Fig. 1e).

### PTPN14 co-immunoprecipitates with CAV1 in the presence of E-cadherin

**Fig. 2:**
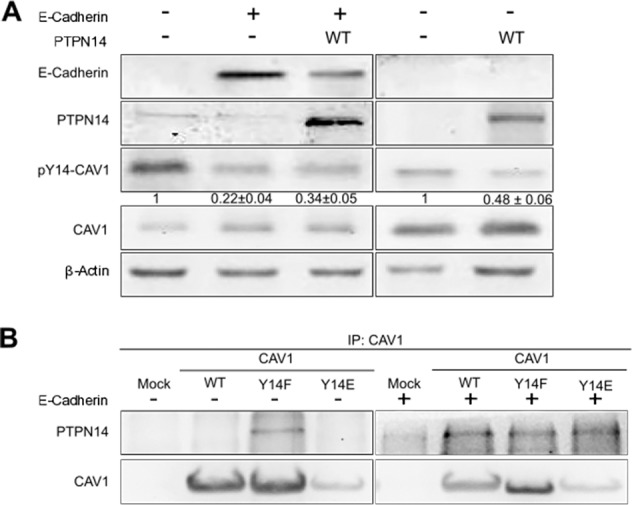
PTPN14 co-immunoprecipitates with caveolin-1 in the presence of E-cadherin. **a** B16F10(CAV1) or B16F10(CAV1-Ecadherin) cells were transfected with 2 μg of pcDNA3-V5-PTPN14-wild-type plasmid for the expression of PTPN14. Proteins (50 µg) were then separated by SDS-PAGE, transferred to nitrocellulose and the presence of E-cadherin, PTPN14, Caveolin-1 phosphorylated on Tyr-14 (pY14-CAV1), caveolin-1 (CAV1), and β-actin were detected using specific antibodies. Blots representative of results obtained in three independent experiments are shown. **b** B16F10(Mock), B16F10(CAV1), B16F10(Mock-Ecadherin), or B16F10(CAV1-Ecadherin) cells were grown for 48 h in the presence of IPTG (1 mM). CAV1 was immunoprecipitated using a polyclonal antibody against CAV1 (2.5 μg/condition) and extracts containing 2 mg of total protein from B16F10(Mock), B16F10(CAV1/WT), B16F10(CAV1/Y14F), B16F10(CAV1/Y14E), or from the same cells transfected with 2 μg of pBATEM2 plasmid for the expression of E-cadherin. The proteins PTPN14 and CAV1 present in immunoprecipitates were detected by immunoblotting using specific antibodies. Western blots representative of results obtained in 3 independent experiments are shown.

Since E-cadherin co-immunoprecipitated with CAV1 and decreased CAV1 phosphorylation on Y14 in B16F10 cells, we hypothesized that the multiprotein complex formed by CAV1 and E-cadherin may contain a specific tyrosine phosphatase responsible for CAV1 dephosphorylation. To identify this candidate protein, we immunoprecipitated CAV1 from B16F10(CAV1) cells lacking E-cadherin or the same cells expressing E-cadherin [B16F10(CAV1-E-cadherin)] and subjected the resulting multiprotein complexes to analysis by mass spectrometry. The proteins that co-immunoprecipitated with CAV1 were initially trypsin digested, then the resulting peptides were analyzed by mass spectrometry and the obtained peptide fragments were compared using the database Mascot. According to those results, the only phosphatase detected in the CAV1 immunoprecipitates (from cells expressing E-cadherin) was the non-receptor type 14 protein tyrosine phosphatase (PTPN14) (Table 1). To corroborate these observations, the presence of PTPN14 was confirmed by Western blotting in B16F10 cells expressing or not CAV1 (Fig. 2a), as well as in the human gall bladder cancer cells Gbd1 (Supplementary Fig. 2a) and the colon cancer cell line DLD-1 (Supplementary Fig. 2b) that endogenously express E-cadherin, PTPN14, and CAV1. In addition, the effect of PTPN14 overexpression on Y14-CAV1 phosphorylation in the absence or presence of E-cadherin was determined. The overexpression alone of PTPN14 decreased Y14-CAV1 phosphorylation by about 50% while the co-expression of E-cadherin and PTPN14 reduced phosphorylation by almost 70% (Fig. 2b). These observations indicate that while E-cadherin facilitates PTPN14 access to CAV1, it does not appear to be absolutely necessary.

**Table 1:** PTPN14 co-immunoprecipitates with caveolin-1 in the presence of E-cadherin.

B16F10 (CAV1)	B16F10 (CAV1-E-Cadherin)
Low-density lipoprotein receptor-related protein 2	Multidomain presynaptic cytomatrix protein
Histone acetyltransferase KAT6A	Titin
Tyrosine-protein kinase 1	TopBP1-interacting checkpoint and replication regulator
Protein 4732456N10Rik	Chromodomain-helicase-DNA-binding protein 2
Pleckstrin homology domain-containing family H member 2	Adenomatosis polyposis coli
Amyloid beta A4 precursor protein-binding family B member 2	Fatty acid synthase
Spermatogenesis-associated protein 17	Smad1
RNA exonuclease 1 homolog	S1 RNA-binding domain-containing protein 1
Kinectin	Hydrocephalus-inducing protein
Sorting nexin 14	NAD(P) transhydrogenase, mitochondrial
G-protein coupled receptor 75	RNA-binding protein 48
Voltage-dependent P/Q-type calcium channel subunit alpha-1	Dennd4a, C-myc promoter-binding protein
Oog4 protein	Matrilin-3
Syntaxin-5	Microtubule-actin cross-linking factor 1
Nuclear envelope spectrin repeat protein 2	E3 Ubiquitin-protein ligase RNF213
Magi3, Membrane associated guanylate kinse, WW and PDZ domain containing 3	Serine/threonine-protein kinase WNK2
Protein Atp2b1	Disheveled-associated activator of morphogenesis 2
Acetylcholinesterase collagenic tail peptide	Protocaherin-17
Chaperone activityof bc1 complex-like, mitochondrial	Serine/threonine-protein phosphatase 4 regulatory subunit 3B
Zinc finger CCCH domain-containing protein 6	Ubiquitin carboxyl-terminal hydrolase
Hepatocyte growth factor-regulated tyrosine kinase substrate	Protein Cyp2j 12
Nuclin	Serine/arginine repetitive matrix protein 2
mRNA-decapping enzyme 1B	Protein spindly
Eukaryotic translation initiation factor 4E type 1B	DIP2 disco-interacting protein 2 homolog C
E3 ubiquitin -protein ligase MYCBP2	PH-interacting protein
Chromodomain-helicase-DNA-binding protein 9	MLX-interacting protein
Zinc finger protein 800	Diaphanous homolog 3
	Centriolin
	Cyclin B3
	HEAT repeat-containing protein 3
	Kinesin-like protein KIF9
	Multiple PDZ domain protein
	FAM129C, Phospholipid binding
	Serine (or cysteine) peptidase inhibitor, clase B, member 9b
	Nucleosome -remodeling factor subunit BPTF
	Metabotropic glutamate receptor 1
	Latrophilin-1
	Afadin
	Bdh2, 3-hydroxbutyrate dehydrogenase type 2
	Mitochondrial peptide methionine sulfoxide reductase
	Protein Jumonji
	DNA repair and recombination protein RAD54B
	DnaJ homolog subfamily C member 7
	Nucleolar RNA helicase 2
	Citron Rho-interacting kinase
	BOD1L
	**Tyrosine-protein phoshatase non-receptor type 14 (PTPN14)**
	BADHD1, Bromo adjacent homology domain-containing 1 protein

B16F10(Mock), B16F10(CAV1), B16F10(Mock-Ecadherin), or B16F10(CAV1-Ecadherin) cells were grown for 48 h in the presence of IPTG (1 mM). CAV1 was immunoprecipitated in each case using the polyclonal antibody against CAV1 covalently immobilized on metallic spheres (Dynabeads®). The proteins that co-immunoprecipitated with CAV1 were eluted and digested with trypsin. The resulting peptides were analyzed in a capillary reverse-phase chromatographer with an electrospray coupled to a mass spectrometer (MS/MS) and an ion trap. The possible modifications, such as oxidation of methionine or an incomplete trypsin digest were considered. Data shown were selected using a probability higher than 0.9 for peptides and 0.95 for proteins. The table shows the results of 3 independent experiments in triplicate plus an analytical triplicate. Only the unique proteins and peptides found in B16F10(CAV1) (left column) and B16F10(CAV1-Ecadherin) (right column) were tabulated.

The CAV1 immunoprecipitates were also characterized by Western blotting in B16F10 cells that expressed either wild type (CAV1), a non-phosphorylatable [CAV1(Y14F)] or a phosphomimetic [CAV1(Y14E)] CAV1 protein, in the absence or presence of E-cadherin (Fig. 2b). In the absence of E-cadherin, PTPN14 only co-immunoprecipitated with CAV1 in those cells expressing the non-phosphorylatable CAV1(Y14F) mutant protein, which appears to act as a PTPN14 trapping mutant (Fig. 2b, left panel). However, in cells that expressed E-cadherin, PTPN14 co-immunoprecipitated with all forms of CAV1 (Fig. 2b, right panel). These observations again suggest that in cells expressing E-cadherin, a complex is formed with CAV1 that more effectively recruits the phosphatase PTPN14 and is likely responsible for decreasing CAV1 phosphorylation on Y14. Importantly, no previous reports are available in the literature implicating CAV1 as a substrate for PTPN14.

### PTPN14 expression inhibits CAV1-enhanced migration and invasion of metastatic cancer cells through Rac-1 inhibition

**Fig. 3:**
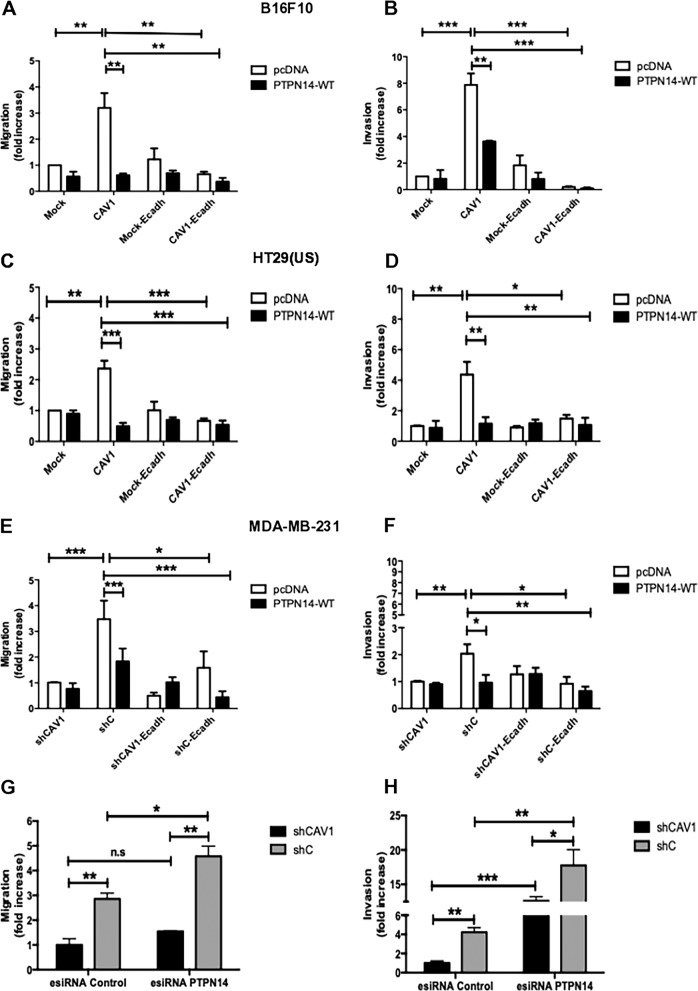
PTPN14 expression inhibits caveolin-1-enhanced cell migration and invasion of metastatic cancer cells. **a**, **b** B16F10(Mock), B16F10(CAV1), B16F10(Mock-Ecadherin), B16F10(CAV1-Ecadherin), **c**, **d** HT29(US)(Mock), HT29(US)(CAV1), HT29(US)(Mock-Ecadherin) or HT29(US)(CAV1-Ecadherin), **e**, **f** MDA-MB-231(shCAV1), MDA-MB-231(shC), MDA-MB-231(shCAV1-Ecadherin), cells were transfected with 2 μg of pcDNA3-V5-PTPN14-WT plasmid (PTPN14-WT) for the expression of PTPN14 phosphatase or with the empty vector (pcDNA3) as a control. **g**, **h** MDA-MB-231(shC) and MDA-MB-231(shCAV1) cells (6 × 10^5^) were transfected with 5 μM of siRNA against PTPN14 (EHU054171, Sigma-Aldrich) or with an siRNA control. **a**, **c**, **e**, **g** Cells (2 × 10^5^) were then seeded in Boyden chambers coated with fibronectin (2 μg/ml) and allowed to migrate for 2 h (B16F10 and MDA-MB-231 cells) or for 5 h [HT29(US) cells]. The cells that migrated through the pores were stained and counted. **b**, **d**, **f**, **h** Cells (2 × 10^5^) were seeded in Matrigel coated chambers and allowed to invade the matrix for 24 h. The cells that accumulated on the lower surface of the membrane were then stained and counted. Values obtained were normalized to those that do not express CAV1 [Mock for HT29(US) and B16F10] or shCAV1 (for MDA-MB-231) cells. The graphs show the average of results from 3 independent experiments (mean ± SEM). Significant differences are indicated ***p < 0.001, **p < 0.01, *p < 0.05.

Previous studies from our laboratory in murine melanoma, metastatic breast, and colon cancer cells showed that the expression of CAV1 favors cell migration in 2D [4, 5]. Alternatively, the expression of E-cadherin suppresses the ability of CAV1 to increase migration of melanoma cells in vitro and promote metastasis in vivo [5]. Hence, given that in the presence of E-cadherin, CAV1 co-immunoprecipitated with PTPN14, we evaluated how overexpression of PTPN14 affected cell migration and invasion induced by CAV1 in metastatic cells. To this end, B16F10(Mock) and (CAV1), HT29(US)(Mock) and (CAV1) and MDA-MB-231(shCAV1) and (shC) cells, expressing or not E-cadherin (E-cadh), were transfected with the plasmid pcDNA3-V5-PTPN14-WT for the expression of PTPN14 or with the empty vector control (pcDNA3.1). In subsequent migration and invasion assays, we observed that CAV1-enhanced cell migration was inhibited by the expression of E-cadherin in all three metastatic cell lines (Fig. 3a, c, e, white bars). Likewise, a similar outcome was observed in invasion assays (Fig. 3b, d, f, white bars). Interestingly, overexpression of PTPN14 alone sufficed to suppress CAV1-enhanced cell migration (Fig. 3a, c, e, black bars) and invasion (Fig. 3b, d, f, black bars). Alternatively, the silencing of PTPN14 in metastatic breast cancer cells that express [MDA-MB-231(shC)] or not CAV1 [MDA-MB-231(shCAV1)] using an esiRNA against PTPN14 (Supplementary Fig. 1b) increased cell migration (Fig. 3g), as well as invasion of those MDA-MB-231 cells expressing CAV1 (Fig. 3h).

To assess which segment(s) of the PTPN14 protein participated in the inhibition of CAV1-enhanced migration and invasion, B16F10 cells were transfected with the plasmid encoding the full-length wild type PTPN14 or PTPN14 mutants that lack either the N-terminal (FERM domain) or the C-terminal domain (catalytic activity) (Supplementary Fig. 1c). Then, CAV1 was immunoprecipitated and the phosphorylation status evaluated with the antibody specific for tyrosine-14 phosphorylated CAV1. For the wild type and N-terminal PTPN14 deletion constructs (harboring the phosphatase domain), CAV1 phosphorylation on tyrosine-14 was clearly reduced in comparison to the level observed when co-expressing the C-terminal deletion (lacking the phosphatase domain) (Supplementary Fig. 1d), indicating that the catalytic activity of PTPN14 is required to dephosphorylate CAV1 tyrosine-14.

**Fig. 4:**
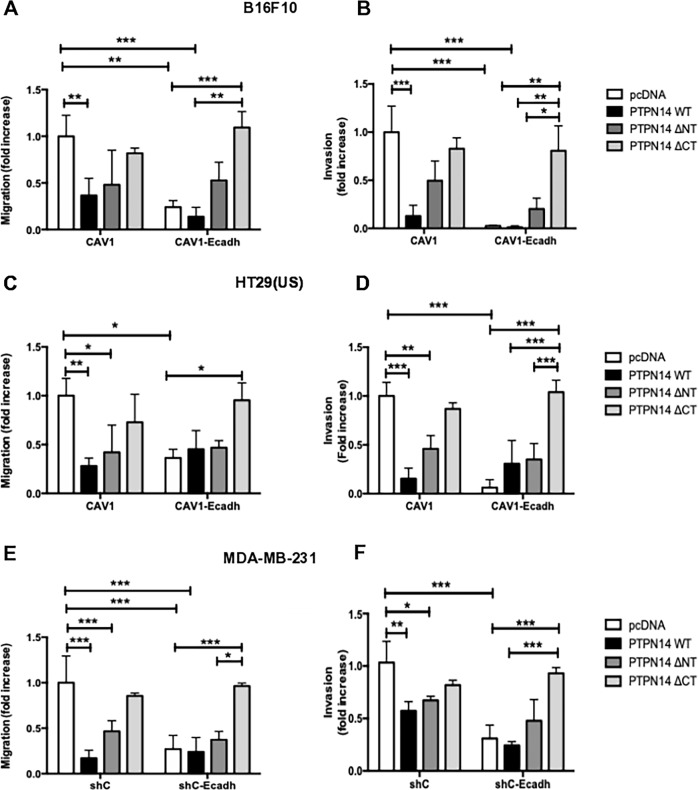
The lack of phosphatase activity eliminates the migration and invasion suppressive effect of E-cadherin upon caveolin-1 in metastatic cells. **a**, **b** B16F10(CAV1), B16F10(CAV1-Ecadherin), (**c**, **d**) HT29(US)(CAV1), HT29(US)(CAV1-Ecadherin), (**e**, **f**) MDA-MB-231(shC), MDA-MB-231(shCAV1-Ecadherin), cells were transfected with 2 μg of pcDNA3-V5-PTPN14-WT (PTPN14 WT), pcDNA3-V5-PTPN14-del-N (PTPN14 ΔNT) or pcDNA3-V5-PTPN14-del-C (PTPN14 ΔCT) plasmids for the expression of PTPN14 phosphatase wild type or its truncated versions lacking the N or C-terminal domain respectively or with the empty vector (pcDNA3) as a control. **a**, **c**, **e** Cells (2 × 10^5^) were then seeded in Boyden chambers coated with fibronectin (2 μg/ml) and allowed to migrate for 2 h (B16F10 and MDA-MB-231 cells) or for 5 h (HT29(US) cells). The cells that migrated through the pores were stained and counted. **b**, **d**, **f** Cells (2 × 10^5^) were seeded in Matrigel coated chambers and allowed to invade the matrix for 24 h. The cells that accumulated on the lower surface of the membrane were then stained and counted. Values obtained were normalized to those that express CAV1 [for HT29(US) and B16F10] or shC [for MDA-MB-231] cells. The graphs show the average of results from 3 independent experiments (mean ± SEM). Significant differences are indicated ***p < 0.001, **p < 0.01, *p < 0.05.

Using these same cells, migration and invasion assays were performed (Fig. 4). Transfection of the B16F10 cells with the construct encoding full-length PTPN14 significantly reduced CAV1-induced migration (Fig. 4a, c, e, Supplementary Table 1) and invasion (Fig. 4b, d, f, Supplementary Table 1). This was generally also the case for the PTPN14 construct lacking the N-terminal region, albeit to a lesser extent than the full-length protein, while PTPN14 lacking catalytic activity had no significant effect even in presence of E-cadherin, which may be taken to suggest that this mutant with the FERM domain displaces the endogenous PTPN14 from the complex. However, additional experiments will be required to show that this is indeed the case.

**Fig. 5:**
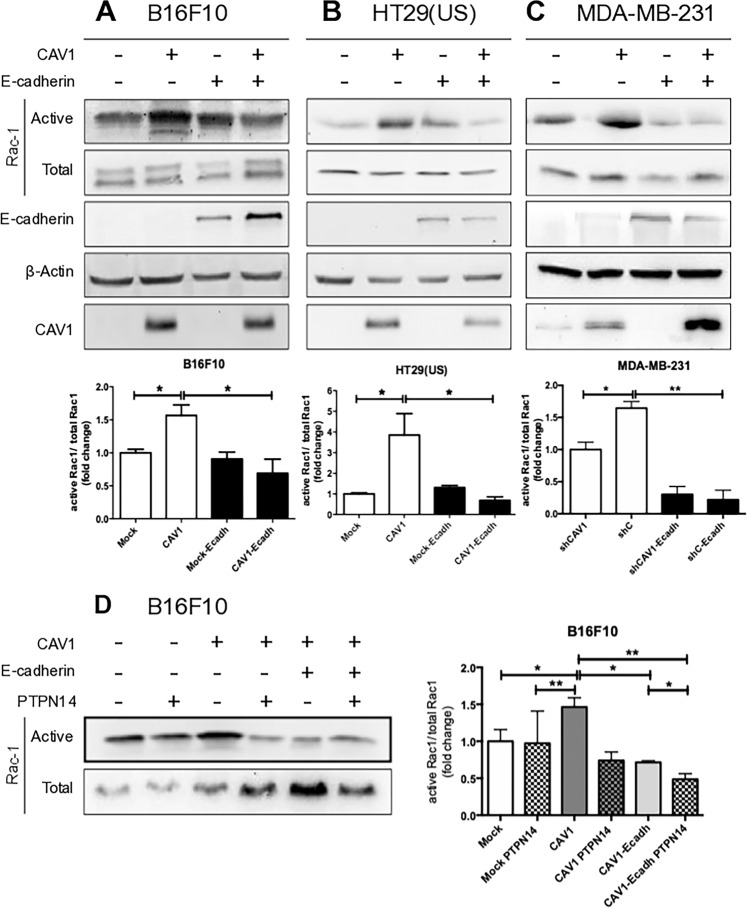
PTPN14 expression inhibits Rac-1 activation induced by caveolin-1 in metastatic cells. **a** B16F10(Mock) and B16F10(CAV1), **b** HT29(US)(Mock) and HT29(US)(CAV1), or **c** MDA-MB-231(shCAV1) and MDA-MB-231(shC), cells (6 × 10^5^) were seeded in 10 cm dishes and 24 h later were transfected with 6 μg of pBATEM2 plasmid, for the expression of E-cadherin or **d** B16F10(Mock), B16F10(Mock-E-cadh), B16F10(CAV1) or B16F10(CAV1-E-cadh) cells were transfected with 2 μg of pcDNA3-V5-PTPN14-wild-type plasmid for the expression of PTPN14. Cells were lysed and supernatants were used immediately for Rac-1 pull-down assays with the fusion protein GST-PBD. Samples were separated by SDS-PAGE (12% acrylamide) and analyzed by Western blotting. Results obtained by western blotting were quantified by scanning densitometry. The graphs show the means from 3 independent experiments (mean ± SEM) of the ratio Active Rac-1/Total Rac-1 normalized to the control condition (Mock). Statistically significant differences are indicated **p < 0.01, *p < 0.05.

In metastatic cancer cells, activation of the small GTPase Rac-1 [4, 13], required for CAV1 to promote cell migration and invasion, involves upstream activation of Rab-5, another small GTPase [13]. E-cadherin expression significantly decreased Rac-1 activation in B16F10(CAV1) (Fig. 5a), HT29(US)(CAV1) (Fig. 5b), and MDA-MB-231(shC) (Fig. 5c) cells, as well as Rab-5 activation in B16F10 and HT29(US) cells (Supplementary Fig. 3a and b). No such effect was seen in B16F10(Mock), HT29(US)(Mock), and MDA-MB-231(shCAV1) cells that express little or no CAV1 (Fig. 5 and Supplementary Fig. 3). These observations can be taken to suggest that recruitment of PTPN14 to the complex formed by CAV1 and E-cadherin is important for subduing the ability of CAV1 to activate Rac-1. Indeed, the introduction of PTPN14 into B16F10 cells that expressed CAV1 in the absence of E-cadherin sufficed to decrease Rac-1; however, in cells that co-expressed CAV1 and E-cadherin, the additional expression of PTPN14 potentiated Rac-1 inhibition (Fig. 5d). Therefore, the recruitment of PTPN14 to the multiprotein complex formed by CAV1 and E-cadherin inhibits cell migration and invasion by preventing CAV1-dependent Rac-1 activation.

### PTPN14 expression suppresses CAV1-enhanced metastasis in vivo

**Fig. 6:**
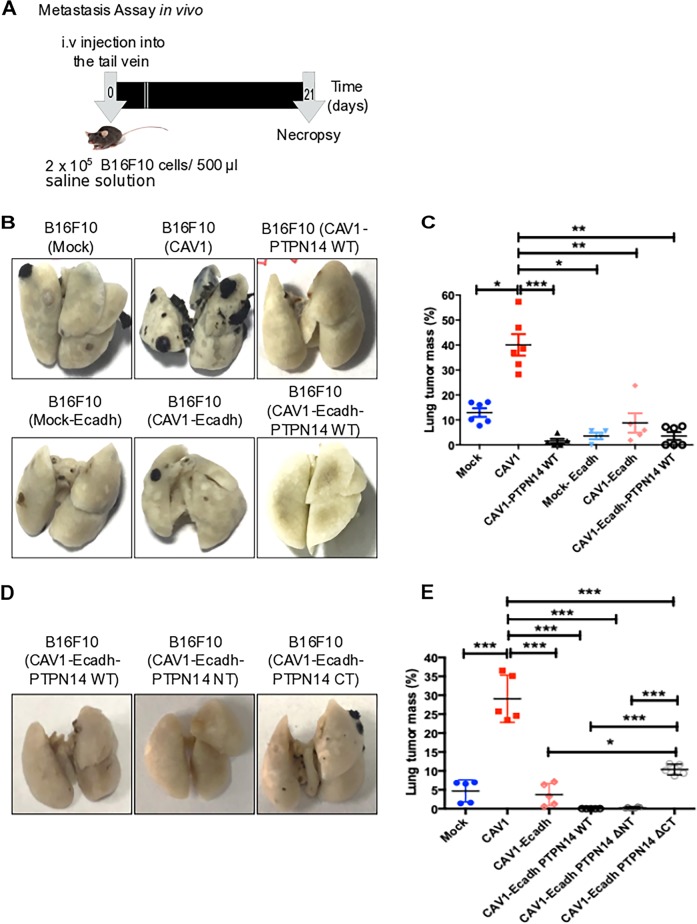
PTPN14 expression suppresses the metastasis promoting role of caveolin-1 in vivo. **a** Schematic representation of the experimental design. C57BL/6 mice were injected intravenously into the tail vein with B16F10 cells (2 × 10^5^) that were grown for 48 h in the presence of IPTG (1 mM). After 21 days mice were sacrificed and the complete lung, as well as metastatic tumor mass were determined. **b**, **d** Representative photographs of lung metastasis in each condition. **c** Average values (mean ± SEM) for lung occupation by tumors in C57BL/6 mice inoculated with B16F10(Mock), B16F10(CAV1), B16F10(CAV1-PTPN14 WT), B16F10(Mock-Ecadherin), B16F10(CAV1-Ecadherin), or B16F10(CAV1-Ecadherin-PTPN14 WT) were 13 ± 1.7%, 40 ± 4.3%, 1.5 ± 0.9%, 3.6 ± 1.3%, 8.8 ± 3.8%, 3.6 ± 1.6%, respectively, statistically significant differences are indicated ***p < 0.001, **p < 0.01, *p < 0.05. **e** Average values (mean ± SEM) for lung occupation by tumors in C57BL/6 mice inoculated with B16F10(Mock), B16F10(CAV1), B16F10(CAV1-Ecadherin), B16F10(CAV1-Ecadherin PTPN14 WT), B16F10(CAV1-Ecadherin PTPN14 ΔNT), or B16F10(CAV1-Ecadh PTPN14 ΔCT) were 5 ± 2.9%, 29 ± 6.2%, 3.7 ± 2.9%, 0.02%, 0.3 ± 0.3%, and 10 ± 1.2%, respectively. Statistically significant differences are indicated ***p < 0.001, *p < 0.05.

Overexpression of PTPN14 inhibited cell migration and invasion induced by CAV1 in vitro. Thus, we determined if PTPN14 expression was sufficient to reduce CAV1-enhanced metastasis in vivo. To this end, B16F10(Mock) or (CAV1) cells were transfected either with E-cadherin or PTPN14 alone, or co-transfected with both and then injected (2 ×10^5^) into the tail vein of C57/BL6 mice (Fig. 6a). After 21 days, the mice were sacrificed and the black tumor mass in the lungs of B16F10 cells was visualized (Fig. 6b). The percentage of black tumor mass found, indicated that the expression of CAV1 increased metastasis roughly threefold in comparison to cells that do not express CAV1 (Mock cells: 13% of lung mass) and the expression of E-cadherin decreased the tumor mass to a similar extent (Fig. 6c) as we described previously [5]. The expression of PTPN14 in B16F10(CAV1) cells reduced metastasis due to CAV1 (1.6% of lung mass) to an extent comparable to E-cadherin alone (8.8% of lung mass) or when co-expressed with E-cadherin (3.6% of lung mass) (Fig. 6c). The expression of truncated PTPN14 mutants in vivo was also assessed. In cells that co-express CAV1/ E-cadherin and catalytically active PTPN14 constructs (full-length and N-terminal deletion), metastasis was reduced similarly as for E-cadherin alone. Alternatively, upon co-expression of the PTPN14 construct lacking the C-terminal catalytic domain, inhibition of metastasis due to E-cadherin presence was partially reverted (Fig. 6d and e).

## Discussion

Previous work from our laboratory showed that E-cadherin and CAV1 synergize in suppressing subcutaneous tumor formation by B16F10 cells in syngeneic C57BL/6 mice. In addition, E-cadherin completely suppresses the ability of CAV1 to promote lung metastasis in the same model [5]. In human colon adenocarcinoma cell lines that co-express E-cadherin and CAV1, the two proteins form a protein complex that recruits β-catenin to the plasma membrane decreasing the expression of survivin [10, 12] and cyclooxygenase-2 [11], thereby reducing cell proliferation and survival. Furthermore, CAV1 and E-cadherin co-expression also reduce survivin expression and enhance apoptosis in human melanoma cells [5]. Thus, while the mechanism by which E-cadherin and CAV1 synergize in tumor suppression is well defined, it remained unclear how E-cadherin blocks the ability of CAV1 to promote migration, invasion and metastasis.

Phosphorylation of CAV1 on Y14 is relevant in this context and has been related to increases in anchorage-independent growth, Grb7-dependent cell migration [34], metalloproteinase activation, and cell invasion [35], membrane microdomain internalization regulated by integrins [26] as well as caveolae formation induced by EGF [36]. Also, CAV1 expression is associated with an increase in MDA-MB-231 and B16F10 cell migration [4], which is blocked by inhibition either pharmacologically of Src family kinases with PP2 or using the non-phosphorylatable CAV1(Y14F) mutant [4, 7]. Here we show that E-cadherin inhibited Y14-CAV1 phosphorylation and co-immunoprecipitated with CAV1, recruiting to this complex PTPN14, which has a well described role during development [37]. Deficiency or loss of PTPN14 expression induces defects in zebrafish [38] and Drosophila [39] development, through the activation of LATS1, a serine/threonine kinase that regulates the Hippo signaling pathway [40]. In HeLa cells, PTPN14 overexpression alters the cell morphology and actin cytoskeleton, thereby reducing cell–matrix adhesion [41, 42]. PTPN14 knockdown in breast cancer cells or the intraperitoneal injection of mice with conditioned media from PTPN14-knockdown cells, promotes the growth and metastasis of breast cancer xenografts [32]. Thus, while PTPN14 clearly functions as a tumor and metastasis suppressor, little information is available about relevant PTPN14 targets.

To date five PTPN14 substrates have been identified; β-catenin [43], YAP [44], p130Cas [31], RIN1, and PKC-δ [32], which are all involved in tumor progression and metastasis. Therefore, the characterization of new substrates of this phosphatase is important to understand the role of PTPN14 in cancer progression. This study identifies CAV1 as a novel substrate for PTPN14, since the phosphatase co-immunoprecipitates with CAV1 in murine melanoma cells B16F10 that express E-cadherin and over expression of PTPN14 reduces the phosphorylation of CAV1 on Y14 (Fig. 2a).

CAV1 co-immunoprecipitation with PTPN14 was generally not observed in the absence of E-cadherin, with the exception of the CAV1(Y14F) mutant protein (Fig. 2). This is to be expected because tyrosine phosphatases typically only interact transiently with their substrates [31]. The dependence on E-cadherin for stable interaction is likely explicable by the fact that PTPN14 binds to E-cadherin rather than CAV1, possibly due to the presence of a FERM domain in the protein [31, 38, 43]. However, we did observe co-immunoprecipitation of CAV1(Y14F) with PTPN14 in the absence of E-cadherin, which is indicative of a direct interaction between the two proteins. Furthermore, only overexpression of PTPN14 constructs containing the catalytic domain were able to reduce the levels of CAV1 phosphorylation on tyrosine-14 (Supplementary Fig. 1d). Taken together, these observations suggest that E-cadherin aids in recruiting PTPN14 to CAV1 proximity, but that E-cadherin presence is not absolutely required for the phosphatase to dephosphorylate pY14-CAV1. However, in situations where PTPN14 expression is relatively low (endogenous expression in cancer cells), E-cadherin expression becomes necessary to promote CAV1 dephosphorylation (Fig. 1) and thereby preclude CAV1-mediated migration, invasion, and metastasis (Fig. 3).

In invasive ductal breast carcinomas, the expression of PTPN14 is lower (threefold) in comparison to ductal carcinomas, and the survival of breast cancer patients decreased if the tumors expressed high levels of the PTPN14 substrates, RIN1 or PKC-δ [32]. We show here for metastatic breast, colon, and melanoma cells that the overexpression of PTPN14 reduces CAV1 pY14, as well as CAV1-induced migration and invasion (Fig. 3) in vitro, and the metastasis promoting role of CAV1 in vivo (Fig. 6). This is the first report linking the tumor suppressor function of PTPN14 to regulation of Y14-CAV1 phosphorylation.

CAV1 favors the activation of the small GTPase Rac-1 in colon, breast, and melanoma cancer cells, which in turn increases cell spreading, cell migration, and invasion [4, 5, 13, 14]. Here, we observed that E-cadherin expression inhibits Rab-5 and Rac-1 activation induced by CAV1 (Supplementary Fig. 3 and Fig. 5a–c). Likewise, PTPN14 expression reduced the activation of Rac-1 induced by CAV1 (Fig. 5d). Thus, PTPN14 controls CAV1 Y14 phosphorylation and thereby limits the ability of the protein to activate these two small G-proteins.

Previous work from our laboratory identified a novel signaling axis in which CAV1 activates Rab-5 by sequestering the GAP protein p85α, thereby promoting Tiam1 recruitment to early endosomes positive for Rab-5 and activating Rac-1 [13, 14]. Interestingly, one of the recently described substrates for PTPN14 is RIN1, a Rab-5 GEF. RIN1 can be phosphorylated on tyrosine 36 by Abl, creating a binding site for Abl through its SH2 domain, thereby releasing the auto-inhibition of Abl, favoring activation of the kinase [45, 46] to increase cell migration and metastasis [47, 48]. It is intriguing to speculate that this sequence of events may also be linked to Y14-CAV1 phosphorylation [49]. Therefore, in cells that express E-cadherin, PTPN14 may dephosphorylate RIN1 on Y16 [32], possibly following recruitment to the multiprotein complex containing CAV1. This may prevent RIN1 from acting as a Rab-5 GEF thereby maintaining Rab-5 in its inactive form (Rab-5-GDP) and promoting the transition of early endosomes to late endosomes, which are degraded by the lysosomal route [32]. As a consequence, Rac-1 would remain inactive (Rac-1-GDP) and this would prevent the increase in migration, invasion, and metastasis induced by CAV1. While highly intriguing, these possibilities need to be evaluated in future experiments.

**Fig. 7:**
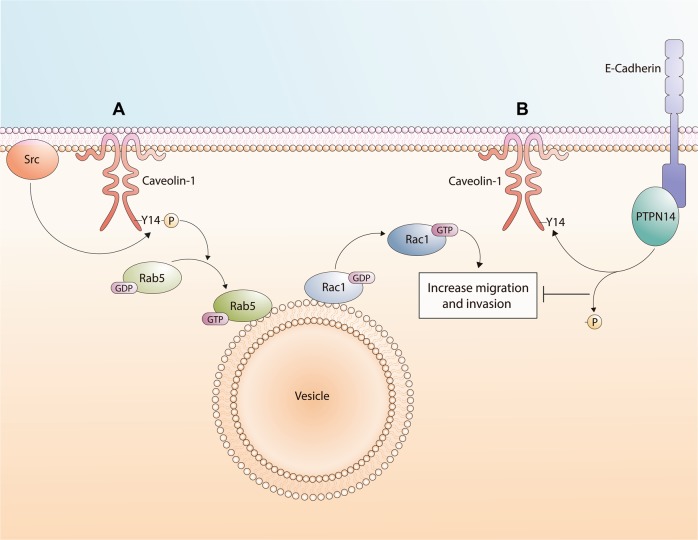
Working model summarizing the main findings described in this study. In metastatic cells lacking E-cadherin, CAV1 phosphorylation on Y14 favors Rac-1 activation, through Rab-5 activation [13], promoting an increase in cell migration, invasion and metastasis. In the presence of E-cadherin, a multiprotein complex is formed with CAV1 that enhances recruitment of the PTPN14 phosphatase, which dephosphorylates pY14-CAV1, inhibiting CAV1-induced Rac-1 activation and the ability of CAV1 to promote migration, invasion and metastasis. However, increasing the expression of PTPN14 wt even in the absence of E-cadherin was sufficient to inhibit CAV1-dependent activation of Rac-1, as well as cell migration, invasion and metastasis in vivo. In conjunction, these results suggest that under conditions where PTPN14 expression is relatively low (endogenous expression), E-cadherin is required to promote efficient pY14-CAV1 dephosphorylation.

In summary (see Fig. 7), for metastatic cells lacking E-cadherin, CAV1 phosphorylation on Y14 favors Rac-1 activation, thereby promoting cell migration, invasion, and metastasis. On the other hand, in the presence of E-cadherin, a multiprotein complex with CAV1 is formed that aids in recruiting PTPN14, which dephosphorylates CAV1 on Y14, inhibiting CAV1-induced Rac-1 activation, and preventing in this way CAV1 from promoting migration, invasion, and metastasis. However, expression of PTPN14 wt alone was sufficient to prevent CAV1-enhanced Rac-1 activation, as well as cell migration, invasion, and metastasis in vivo, suggesting that while E-cadherin potentiates the CAV1/PTPN14 interaction, making it more stable and efficient, this does not represent an absolute prerequisite for CAV1 dephosphorylation by PTPN14. Nonetheless, under conditions where endogenous PTPN14 expression is relatively low, E-cadherin facilitates recruitment to CAV1 proximity, and Y14 dephosphorylation.

## Materials and methods

### Materials

Rabbit polyclonal anti-CAV1, mouse monoclonal anti-CAV1, mouse monoclonal anti-pY14-CAV1, and mouse monoclonal anti-E-cadherin (BD Transduction Laboratories, Lexington, KY, USA), mouse monoclonal anti-PTPN14, and rabbit polyclonal anti-actin (R&D Systems, Minneapolis, MN, USA) antibodies were used as indicated by the manufacturers. Goat anti-rabbit and goat anti-mouse IgG antibodies coupled to horseradish peroxidase (HRP) were from Merck-Millipore (Billerica, Massachusetts, USA) and KPL Laboratories (Washington DC, USA), respectively. The ECL chemiluminescent substrate and the BCA protein determination kit were from Pierce (Rockford, IL, USA). The Plasmid Midi Kit was from Qiagen (Valencia, CA, USA). Human fibronectin was from Becton Dickinson (San Jose, CA, USA). Hygromycin was from Calbiochem (La Jolla, CA, USA). Fetal bovine serum (FBS) was from Biological Industries (Cromwell, CT, USA). Cell culture media and antibiotics were from GIBCO (Invitrogen, Carlsbad, CA, USA), Fugene 6® from Roche (Basel, Switzerland).

### Cell culture and cell lines

Metastatic murine melanoma cells B16F10 (ATCC, #CRL6475, provided by Laurence Zitvogel, Institut Gustav Roussy, Villejuif, France), the human colon cancer cell line DLD-1 (ATCC, #CCL-221) transfected with placIOP-CAV1 (see reference [3]) and gallbladder carcinoma cell line GBd1 (RRID #CVCL_H705) were maintained in RPMI 1640 medium. The colon cancer cell line HT29(US), a metastatic derivative of HT29(ATCC) cells from ATCC (ATCC HTB-38) that we have employed previously [8, 10], were cultured in high-glucose DMEM. MDA-MB-231 (ATCC HTB-26) cells were cultured in DMEM-F12 medium. All media were additionally supplemented with 10% FBS, 100 U/ml of penicillin, and 100 μg/mL of streptomycin sulfate. Cells were cultured at 37 °C in a humidified atmosphere containing 5% CO_2._ HT29(US) and B16F10 cell lines stably transfected with the plasmids pLacIOP (referred to as Mock) and pLacIOP-CAV1 (referred to as CAV1, containing the full-length dog CAV1 sequence, NCBI Reference Sequence: NP_001003296.1), as well as MDA-MB-231 cells stably transduced with control (shC) or CAV1-specific short hairpin sequences. The oligonucleotide containing shRNA candidates for CAV1 (#1 CCAGTTAGATTTAGGGATTTA; #2 CCGCTTGTTGTCTACGATCTT; #3 CGACGTGGTCAAGATTGACTT; #4 TGAAGCTATTGGCAAGATATT; #5 GCTTCCTGATTGAGATTCAGT) obtained from the Broad Institute, Cambridge, USA, were described previously [3, 4, 10]. The results shown here were obtained using cells stably transduced with the shCAV1#5 construct. Stably transfected B16F10(CAV1/Y14F) and B16F10(CAV1/Y14E) cells were also previously described [7]. Stable cell lines were selected and maintained in culture medium containing 2 μg/ml puromycin. For the expression of E-cadherin the cells were transiently transfected with the plasmid pBATEM2 (lacking a selection marker), encoding murine E-cadherin under the control of an actin promoter (provided by Amparo Cano, Universidad Autónoma de Madrid, Madrid, Spain). For the expression of PTPN14-WT or PTPN14 lacking either the N-terminal (ΔNT, containing the phosphatase activity domain) or C-terminal domain (ΔCT, containing the FERM domain), the cells were transiently transfected with the plasmids pcDNA3-V5-PTPN14-wild type (#61003), pcDNA3-V5-PTPN14-del-N (#61004) or pcDNA3-V5-del-C (#61005) purchased from Addgene. For the PTPN14-knockdown experiments, MDA-MB-231 cells were transfected transiently with specific esiRNA (EHU054171, Sigma-Aldrich) or an esiRNA control (EHUEGFP, Sigma-Aldrich).

### Western blotting

Cells were rinsed and harvested in ice-cold PBS containing 1 mM orthovanadate, 10 μg/ml benzamidine, 2 μg/ml antipain, 1 μg/ml leupeptin, and 1 mM phenylmethyl-sulphonylfluoride (Ova-BAL-PMSF). Cells were then centrifuged at 3000 × *g* for 2 min at 4 °C and the respective cell pellets were lysed by sonication in extraction buffer (20 mM Hepes pH 7.4, 0.1% NP-40, and 0.1% SDS plus Ova-BAL-PMSF). Protein concentrations in extracts was determined using the BCA protein assay kit. Protein samples were separated by SDS-PAGE (50 μg/lane), transferred to nitrocellulose, blocked in PBS containing 5% non-fat milk and probed overnight at 4 °C with anti-CAV1 (1:5000), anti-E-cadherin (1:3000) or anti-PTPN14 (2 μg/ml) antibodies diluted in PBS or blocked in PBS containing 10% gelatin and 1% Tween-20 and probed overnight at 4 °C with anti-pY14-CAV1 (1:300). Equal protein loading in each lane was confirmed by probing with an anti-β-actin antibody (1:5000). Goat anti-rabbit IgG antibodies coupled to HRP were used to detect bound first antibodies by EZ-ECL. Protein bands were quantified by densitometric analysis using the ImageJ 1.34 s software (available from NIH at http://rsb.info.nih/ij/).

### Multiple wounding assays

The protocol employed was adapted from Chiang et al. [50]. Cells (6 × 10^5^) were seeded in 6 cm plates and allowed to grow until they formed a monolayer of ~80% confluence. Then multiple wounds were introduced with a steel comb (tips of 0.35–0.40 mm and a distance between the tips of 0.6–0.7 mm) such as to cover more than 50% of the initial total surface. The cell monolayer was washed with PBS before adding either serum free media (time 0) or medium containing 3% serum to stimulate migration for different times.

### Migration and invasion assays

Cell migration was evaluated in Boyden Chamber assays (Transwell Costar, 6.5-mm diameter, 8-mm pore size), whereas invasion was evaluated in Matrigel assays (BD Biosciences, 354480), as reported previously [8, 13].

### Immunoprecipitation assays

CAV1 immunoprecipitation was performed using Dynabeads® coupled with protein A (Novex, life technologies) according to the manufacturer’s specifications. Briefly, 2.5 μg of polyclonal anti-CAV1 antibody diluted in 200 μl of PBS-Tween 0.1% were incubated with 50 μl of metallic beads for 10 min at room temperature in a rotating shaker. Then, the beads were separated using a magnet and the solution was discarded. Subsequently, 2 mg of proteins in 500 μl of PBS-Tween 0.1% were incubated for 2 h at room temperature with the beads coupled to the anti-CAV1 antibody in a rotating shaker. The metallic beads were separated, washed three times with PBS and then 70 μl of loading buffer were added to solubilize complexes for analysis by western blotting or the complexes on the beads were digested with trypsin for subsequent peptide analysis by mass spectrometry.

### Analysis of CAV1 immunoprecipitates by mass spectrometry

Solubilized immunoprecipitates (50 μl) plus 44 μl NH_4_HCO_3_ 50 mM were incubated with 1 μl of 0.5 M dithiothreitol (DTT) at 56 °C for 20 min. Then 2.7 μl of 0.55 M iodoacetamide was added and the mixture was incubated in the dark for 15 min. These samples (5 μl) were digested with 2 μl of 1 μg/μl trypsin (Trypsin Gold, Mass Spectrometry Grade, Promega) at 37 °C overnight. Tryptic digests were subjected to reverse-phase separation followed by nano-ESI-MS/MS on a LTQ ion trap as described [51]. The proteins and peptides obtained were compared using the Mascot database and the Thermo Scientific Proteome Discoverer Software.

### Pull-down assays

Pull-down assays were performed as described [13]. Briefly, cells were lysed in a buffer containing 25 mM HEPES, pH7.4, 100 mM NaCl, 5 mM MgCl2, 1% NP-40, 10% glycerol, 1 mM DTT, and protease inhibitors. Extracts were clarified by centrifugation (10,000 × *g* for 1 min at 4 °C). Supernatants were used immediately for pull-down assays. Glutathione beads were pre-coated with 50 μg of either GST-PBD (for Rac-1) or GST-R5BD (for Rab-5) by incubating for 1 h, at 4 °C on a rotating shaker. Pull-down assays were carried out by incubating fresh extracts with 50 μl of pre-coated beads for 15 min at 4 °C on a rotating shaker. Thereafter, beads were washed three times with lysis buffer containing 0.01% NP-40 and protease inhibitors. Samples were separated by SDS-PAGE (12% acrylamide) and analyzed by Western blotting. Results were quantified by scanning densitometry. The fraction of active Rab5 or Rac1 is calculated by normalizing to total protein input for the respective proteins as described previously [13].

### Metastasis assays

All animal experiments were conducted in accordance with the guidelines of CONICYT, Chile and approved by the Bioethics Committee of Facultad de Medicina of Universidad de Chile (CBA #0836 FMUCH). C57BL/6 mice of 8–12 weeks of age were injected intravenously with 2 × 10^5^ B16F10 (Mock), B16F10 (CAV1) cells co-expressing or not E-cadherin, full-length PTPN14 or deletions thereof, in 500 μl physiological saline, then sacrificed on day 21 post injection. Lungs were fixed in Feketes solution (Current Protocols in Immunology, 2000). Black tissue was separated from the rest of the lung and weighed. Metastasis was expressed as black tissue mass/total lung mass in percent post-fixation.

### Statistical analysis

All data are expressed as mean ± standard error of mean (SEM) of three independent experiments. Data were analysed using the non-parametric Kruskal–Wallis test for multiple comparisons with a post test of Dunn or Tukey. Significance (*p* value) was set at the nominal level of *p* < 0.05 or less. All data were processed using INSTAT v 3.05 (GraphPad Software, San Diego, USA, http://www.graphpad.com).

## Supplementary information

Supplementary Figure Legends

Supplementary Figure 1

Supplementary Figure 2

Supplementary Figure 3

Supplementary Table 1
