# Supplementary material for: The non-receptor tyrosine phosphatase type 14 blocks caveolin-1-enhanced cancer cell metastasis
Source: Oncogene. 2020 Mar 9;39(18):3693–709. doi: 10.1038/s41388-020-1242-3 (PMC7190567; doi:10.1038/s41388-020-1242-3)
Supplement: Supplementary file 1 — Supplementary Figure Legends [file 41388_2020_1242_MOESM1_ESM.docx]

**Supplementary Figure 1. Characterization of metastatic cell lines employed in the study.** (**A**) Metastatic murine melanoma B16F10 cells and human colon carcinoma HT29(US) cells that do not express caveolin-1, were stably transfected with either the plasmid pLacIOP (Mock) and pLacIOP-CAV1 (containing the CAV1 sequence). The human breast cancer cells MDA-MB-231 that express endogenously high levels of caveolin-1 were stably transduced with either a control (shC) or a CAV1-specific short hairpin sequence. (**B**) MDA-MB-231(shC) and MDA-MB-231(shCAV1) cells (6 x 10^5^) were seeded in 6 cm dishes and 24 h later were transfected with 5 µM of siRNA against PTPN14 (EHU054171, Sigma-Aldrich) or with an siRNA control. B16F10 cells were transiently transfected with the plasmids pcDNA3-V5-PTPN14-wild type, pcDNA3-V5-PTPN14-del-N or pcDNA3-V5-del-C for the expression of PTPN14-wt or PTPN14 lacking either the N-terminal (ΔNT, containing the FERM domain) or C-terminal (ΔCT, containing the phosphatase activity domain) domain, respectively and (**C**) cells were lysed and proteins (50 µg) were then separated by SDS-PAGE, transferred to nitrocellulose and the presence of E-cadherin, PTPN14, Caveolin-1 and β-actin were detected using specific antibodies or (**D**) Cells were lysed and CAV1 was immunoprecipitated from cell lysates (2 mg total protein) using the polyclonal antibody against CAV1 covalently immobilized on metallic spheres (Dynabeads**®**). Immunoprecipitated CAV1 and associated proteins were then separated by SDS-PAGE, transferred to nitrocellulose and the presence of CAV1 phosphorylated on tyrosine 14 (pY14-CAV1) as well as CAV1 was detected in the immunoprecipitates using specific antibodies.

**Supplementary Figure 2. PTPN14/ E-cadherin/ CAV1 form a complex in other cancer cell lines.** CAV1 was immunoprecipitated using the polyclonal antibody against CAV1 covalently immobilized on metallic spheres (Dynabeads**®**) from (**A**) the human gallbladder carcinoma cell line GBd1 (endogenous expression of E-cadherin, PTPN14 and Caveolin-1) or (**B**) the human adenocarcinoma cell line DLD-1 (endogenous expression of E-cadherin, PTPN14, transfected with plasmid for Caveolin-1 expression). Cells were lysed and proteins (50 µg) were then separated by SDS-PAGE, transferred to nitrocellulose and the presence of E-cadherin, PTPN14 and caveolin-1 in the immunoprecipitates (IPs) and the original extracts (Input) were detected using specific antibodies.

**Supplementary Figure 3. E-cadherin expression inhibits Rab-5 activation induced by caveolin-1 in metastatic cells. (A)** B16F10(Mock) and B16F10(CAV1), **(B)** HT29(US)(Mock), HT29(US)(CAV1), cells (6 x 10^5^) were seeded in 10 cm dishes and 24 h later were transfected with 6 μg of pBATEM2 plasmid, for the expression of E-cadherin. Cells were lysed and supernatants were used immediately for Rab-5 pull-down assays with the fusion protein GST-R5BD. Samples were separated by SDS-PAGE (12% acrylamide) and analyzed by Western blotting. Results obtained by western blotting were quantified by scanning densitometry. The graphs show the means from 3 independent experiments (mean ± SEM) of the ratio Active Rab-5/Total Rab-5 normalized to the control condition (Mock). Statistically significant differences are indicated ** p < 0.01, * p < 0.05.

**Supplementary Table 1. Statistical analysis of migration and invasion assays shown in Figure 4.** Means ± standard errors of mean (SEM) of three independent migration or invasion experiments with the metastatic cells (**A**) B16F10 (**B**) HT29(US) (**C**) MDA-MB-231 are summarized. Data were analyzed using the non-parametric Kruskal-Wallis test for multiple comparisons with a post test of Tukey. Significant differences are indicated *** p< 0.001, ** p < 0.01, * p < 0.05, non-significant differences are indicated as ns.
