# Supplementary figures and images for: The non-receptor tyrosine phosphatase type 14 blocks caveolin-1-enhanced cancer cell metastasis
Source: Oncogene. 2020 Mar 9;39(18):3693–709. doi: 10.1038/s41388-020-1242-3 (PMC7190567; doi:10.1038/s41388-020-1242-3)

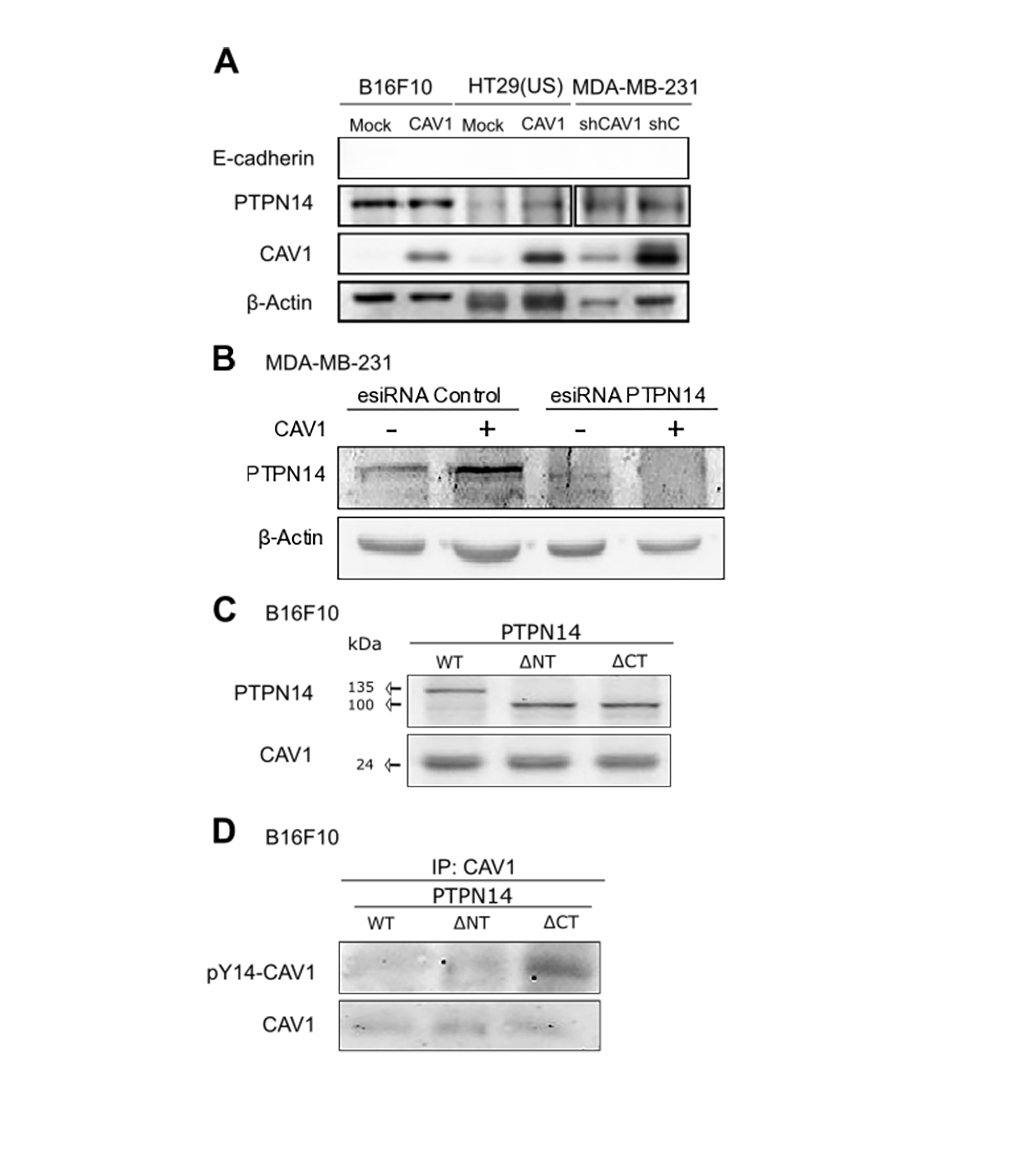

Supplement: Supplementary file 2 — Supplementary Figure 1 [file 41388_2020_1242_MOESM2_ESM.tif]

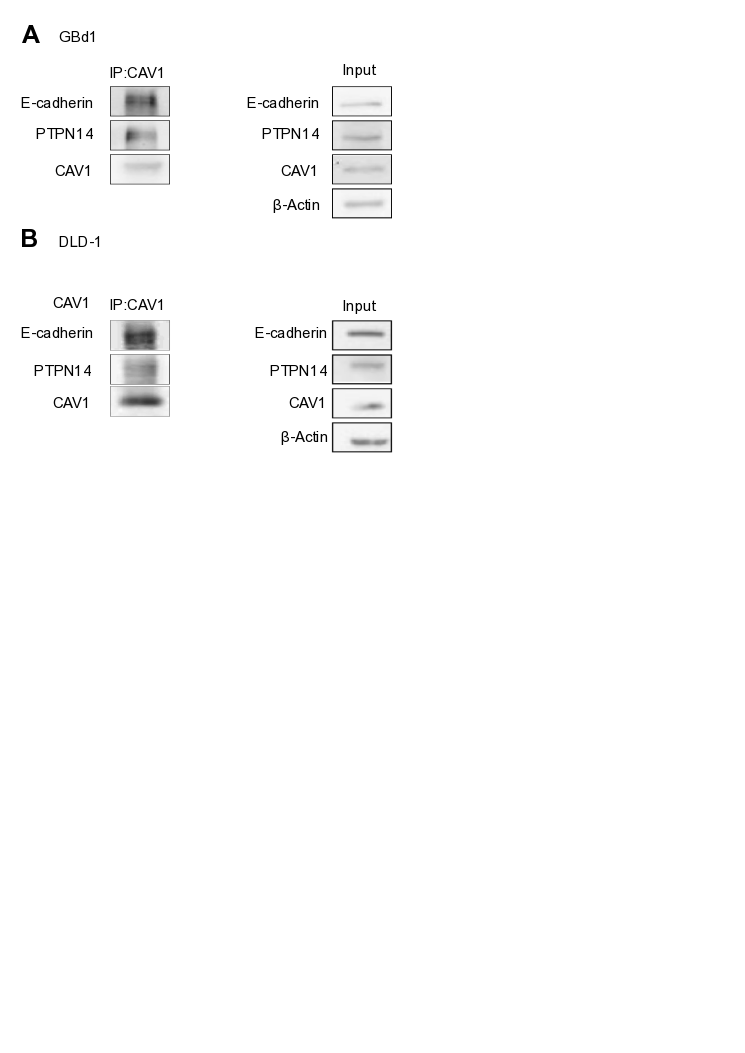

Supplement: Supplementary file 3 — Supplementary Figure 2 [file 41388_2020_1242_MOESM3_ESM.tif]

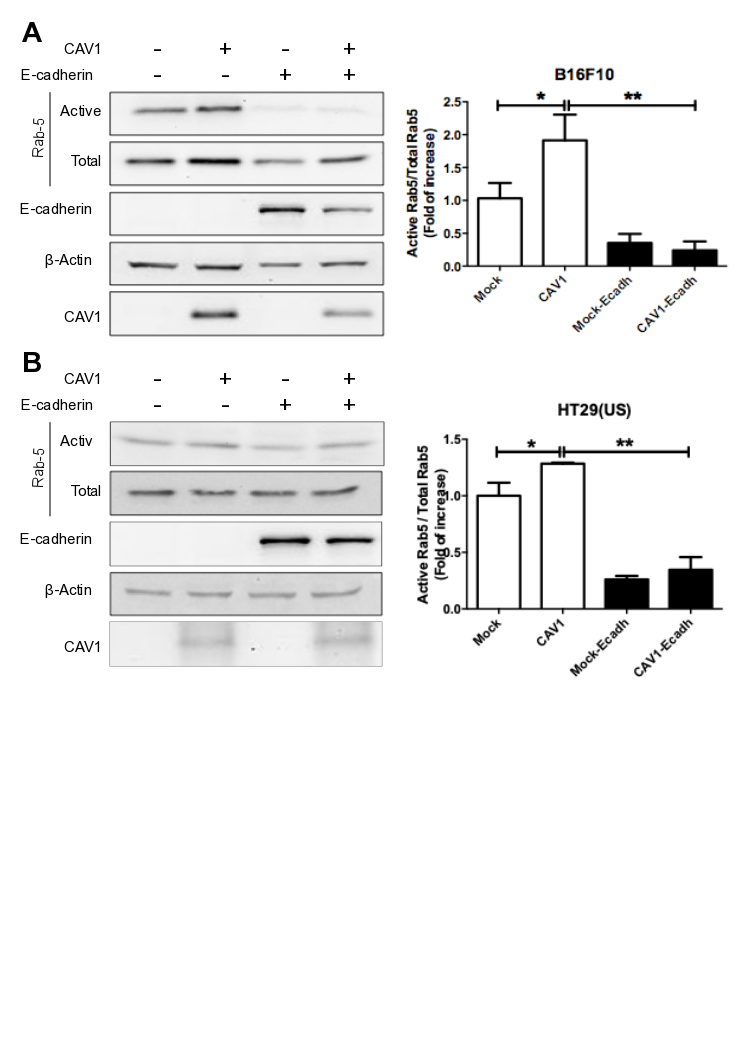

Supplement: Supplementary file 4 — Supplementary Figure 3 [file 41388_2020_1242_MOESM4_ESM.tif]
