# Supplementary material for: The non-receptor tyrosine phosphatase type 14 blocks caveolin-1-enhanced cancer cell metastasis
Source: Oncogene. 2020 Mar 9;39(18):3693–709. doi: 10.1038/s41388-020-1242-3 (PMC7190567; doi:10.1038/s41388-020-1242-3)
Supplement: Supplementary file 5 — Supplementary Table 1 [file 41388_2020_1242_MOESM5_ESM.docx]

|  | 1. **B16F10** | | 1. **HT29(US)** | |
| --- | --- | --- | --- | --- |
|  | **MIGRATION** | **INVASION** | **MIGRATION** | **INVASION** |
| pcDNA CAV1 vs. PTPN14 WT CAV1 | ** | *** | ** | *** |
| pcDNA CAV1 vs. PTPN14 △NT CAV1 | ns | ns | * | ** |
| pcDNA CAV1 vs. PTPN14 △CT CAV1 | ns | ns | ns | ns |
| PTPN14 WT CAV1 vs.PTPN14 △NT CAV1 | ns | ns | ns | ns |
| PTPN14 WT CAV1 vs.PTPN14 △CT CAV1 | ns | ** | ns | *** |
| PTPN14 △NT CAV1 vs. PTPN14 △CT CAV1 | ns | ns | ns | * |
| pcDNA CAV1 vs. pcDNA CAV1-Ecadh | ** | *** | * | *** |
| pcDNA CAV1 vs. PTPN14 WT CAV1-Ecadh | *** | *** | * | *** |
| pcDNA CAV1 vs. PTPN14 △NT CAV1-Ecadh | ns | ** | * | *** |
| pcDNA CAV1 vs. PTPN14 △CT CAV1-Ecadh | ns | ns | ns | ns |
| PTPN14 WT CAV1 vs. pcDNA CAV1-Ecadh | ns | ns | ns | ns |
| PTPN14 WT CAV1 vs. PTPN14 WT CAV1-Ecadh | ns | ns | ns | ns |
| PTPN14 WT CAV1 vs. PTPN14 △NT CAV1-Ecadh | ns | ns | ns | ns |
| PTPN14 WT CAV1 vs. PTPN14 △CT CAV1-Ecadh | ** | ** | ** | *** |
| PTPN14 △NT CAV1 vs. pcDNA CAV1-Ecadh | ns | ns | ns | ns |
| PTPN14 △NT CAV1 vs. PTPN14 WT CAV1-Ecadh | ns | ns | ns | ns |
| PTPN14 △NT CAV1 vs. PTPN14 △NT CAV1-Ecadh | ns | ns | ns | ns |
| PTPN14 △NT CAV1 vs. PTPN14 △CT CAV1-Ecadh | * | ns | * | ** |
| PTPN14 △CT CAV1 vs. pcDNA CAV1-Ecadh | * | ** | ns | *** |
| PTPN14 △CT CAV1 vs. PTPN14 WT CAV1-Ecadh | ** | ** | ns | ** |
| PTPN14 △CT CAV1 vs. PTPN14 △NT CAV1-Ecadh | ns | * | ns | ** |
| PTPN14 △CT CAV1 vs. PTPN14 △CT CAV1-Ecadh | ns | ns | ns | ns |
| pcDNA CAV1-Ecadh vs. PTPN14 WT CAV1-Ecadh | ns | ns | ns | ns |
| pcDNA CAV1-Ecadh vs. PTPN14 △NT CAV1-Ecadh | ns | ns | ns | ns |
| pcDNA CAV1-Ecadh vs. PTPN14 △CT CAV1-Ecadh | *** | ** | * | *** |
| PTPN14 WT CAV1-Ecadh vs. PTPN14 △NT CAV1-Ecadh | ns | ns | ns | ns |
| PTPN14 WT CAV1-Ecadh vs. PTPN14 △CT CAV1-Ecadh | *** | ** | ns | *** |
| PTPN14 △NT CAV1-Ecadh vs. PTPN14 △CT CAV1-Ecadh | * | * | ns | *** |

|  | 1. **MDA-MB-231** | |
| --- | --- | --- |
|  | **MIGRATION** | **INVASION** |
| pcDNA shC vs. PTPN14 WT shC | *** | ** |
| pcDNA shC vs. PTPN14 △NT shC | ** | * |
| pcDNA shC vs. PTPN14 △CT shC | ns | ns |
| PTPN14 WT shC vs. PTPN14 △NT shC | ns | ns |
| PTPN14 WT shC vs. PTPN14 △CT shC | *** | ns |
| PTPN14 △NT shC vs. PTPN14 △CT shC | ns | ns |
| pcDNA shC vs. pcDNA shC-Ecadh | *** | *** |
| pcDNA shC vs. PTPN14 WT shC-Ecadh | *** | *** |
| pcDNA shC vs. PTPN14 △NT shC-Ecadh | ** | *** |
| pcDNA shC vs. PTPN14 △CT shC-Ecadh | ns | ns |
| PTPN14 WT shC vs. pcDNA shC-Ecadh | ns | ns |
| PTPN14 WT shC vs. PTPN14 WT shC-Ecadh | ns | ns |
| PTPN14 WT shC vs. PTPN14 △NT shC-Ecadh | ns | ns |
| PTPN14 WT shC vs. PTPN14 △CT shC-Ecadh | *** | * |
| PTPN14 △NT shC vs. pcDNA shC-Ecadh | ns | * |
| PTPN14 △NT shC vs. PTPN14 WT shC-Ecadh | ns | ** |
| PTPN14 △NT shC vs. PTPN14 △NT shC-Ecadh | ns | ns |
| PTPN14 △NT shC vs. PTPN14 △CT shC-Ecadh | ns | * |
| PTPN14 △CT shC vs. pcDNA shC-Ecadh | ** | ** |
| PTPN14 △CT shC vs. PTPN14 WT shC-Ecadh | ** | *** |
| PTPN14 △CT shC vs. PTPN14 △NT shC-Ecadh | * | * |
| PTPN14 △CT shC vs. PTPN14 △CT shC-Ecadh | ns | ns |
| pcDNA shC-Ecadh vs. PTPN14 WT shC-Ecadh | ns | ns |
| pcDNA shC-Ecadh vs. PTPN14 △NT shC-Ecadh | ns | ns |
| pcDNA shC-Ecadh vs. PTPN14 △CT shC-Ecadh | *** | *** |
| PTPN14 WT shC-Ecadh vs. PTPN14 △NT shC-Ecadh | ns | ns |
| PTPN14 WT shC-Ecadh vs. PTPN14 △CT shC-Ecadh | *** | *** |
| PTPN14 △NT shC-Ecadh vs. PTPN14 △CT shC-Ecadh | ** | ** |
